# Synchrotron multimodal imaging in a whole cell reveals lipid droplet core organization

**DOI:** 10.1107/S1600577520003847

**Published:** 2020-04-23

**Authors:** Frédéric Jamme, Bertrand Cinquin, Yann Gohon, Eva Pereiro, Matthieu Réfrégiers, Marine Froissard

**Affiliations:** a DISCO Beamline, Synchrotron SOLEIL, 91192 Gif-sur-Yvette, France; b Institut Jean-Pierre Bourgin, INRAE, AgroParisTech, Université Paris-Saclay, Versailles 78000, France; c MISTRAL Beamline, ALBA Synchrotron, Cerdanyola del Vallès, Barcelona 08290, Spain

**Keywords:** lipid droplets, label-free imaging, deep UV, fluorescence, transmittance, yeast, synchrotrons, cro-SXT

## Abstract

A lipid droplet (LD) core of a cell consists mainly of neutral lipids, tri­­acyl­glycerols and/or steryl esters (SEs). The structuration of these lipids inside the core is still under debate. Lipid segregation inside LDs has been observed but is sometimes suggested to be an artefact of LD isolation and chemical fixation. LD imaging in their native state and in unaltered cellular environments appears essential to overcome these possible technical pitfalls. Here, imaging techniques for ultrastructural study of native LDs *in cellulo* are provided and it is shown that LDs are organized structures. Cryo soft X-ray tomography and deep-ultraviolet (DUV) transmittance imaging are showing a partitioning of SEs at the periphery of the LD core. Furthermore, DUV transmittance and tryptophan/tyrosine auto-fluorescence imaging on living cells are combined to obtain complementary information on cell chemical contents. This multimodal approach paves the way for a new label-free organelle imaging technique in living cells.

## Abstract 2

Cryo soft X-ray tomography and deep-ultraviolet transmittance imaging reveal partitioning of neutral lipids in a lipid droplet core. Furthermore, deep-ultraviolet transmittance and tryptophan/tyrosine auto-fluorescence multimodal imaging on living cells were combined to obtain complementary information on organelle chemical contents.

## 1. Introduction   

Lipid droplets (LDs) are the lipid-storage organelles in cells (Olzmann & Carvalho, 2019 ▸; Pyc *et al.*, 2017 ▸). LDs receive a lot of attention as their abnormal dynamics are associated with several diseases (obesity, diabetes, atherosclerosis and myo­pathies) (Welte, 2015 ▸). LDs are also promising sources of lipids for the development of derived products, such as biofuels and new molecules for food, medicine and cosmetics (Dyer *et al.*, 2008 ▸). Thus, understanding the dynamics and structure of LDs is of major importance for society. Recent advances in LD biology have revealed that the organelle is much more complex and heterogeneous than expected. Intracellular LD heterogeneity occurs partly because of the evolution of LDs during the life cycle of the organelle, from birth to death, but it also appears to be related to the diversity of LD cellular roles (Thiam & Beller, 2017 ▸; Hashemi & Goodman, 2015 ▸). Furthermore, LDs are in close contact or tightly associated with other organelles such as endoplasmic reticulum (Jacquier *et al.*, 2011 ▸), vacuoles (Bouchez *et al.*, 2015 ▸), peroxisome and mitochondria (Gao & Goodman, 2015 ▸). New rapid non-invasive imaging tools are required for exhaustive studies of unaltered LD dynamics, as well as studies of LD relationships with other intracellular compartments.

On the ultrastructural scale, intra LD heterogeneity is evident, but it is still under debate for the central lipid core (Ohsaki *et al.*, 2014 ▸). LDs consist mainly of neutral lipids, tri­acyl­glycerols (TAGs) and/or steryl esters (SEs), stabilized in the cytoplasmic environment by a phospho­lipid monolayer and some proteins (Kory *et al.*, 2016 ▸). Historically, structural data on central lipid cores were obtained on purified LDs using cryoelectron microscopy (Tauchi-Sato *et al.*, 2002 ▸) or small-angle X-ray scattering (SAXS) (Czabany *et al.*, 2008 ▸). These *ex cellulo* approaches revealed ordered LD cores. Czabany and colleagues proposed an ‘ordered shells’ model, essentially a TAG inner core surrounded by SE concentric layers. Electron microscopy on adipocyte ultrathin sections also revealed core partitioning depending on lipid incorporation (Cheng *et al.*, 2009 ▸). Nevertheless, this lipid segregation inside the lipid core could be an artefact of LD isolation or chemical fixation (Ohsaki *et al.*, 2014 ▸). Preservation of an LD in its native form and in its unaltered cellular environment appears essential to overcome these possible technical pitfalls. Recent technical advances offer this opportunity. Observations of LDs maintained *in cellulo* were performed on 200 nm vitreous lamellae of HeLa cells using cryo-focused ion beam/scanning electron microscopy (cryo-FIB). Concentric rings were observed, reinforcing the model of lipid concentric layers in LD cores (Mahamid *et al.*, 2019 ▸).

To improve our knowledge on LD ultrastructure in a preserved cellular environment, we used imaging techniques that do not require chemical fixation and are suitable for observations of intact cells. We performed observations on *Saccharomyces cerevisiae* to take advantage of this well described model. LDs and all the intracellular compartments are easy to identify using conventional microscopy techniques, and a set of yeasts with contrasted LD morphologies and LD lipid contents are available (Fei *et al.*, 2008 ▸; Sandager *et al.*, 2002 ▸).

First, we performed cryo soft X-ray tomography (cryo-SXT) on vitrified *S. cerevisiae* with a nanometric spatial resolution (30 nm half pitch) in a volume of a few micrometres. This technique is well suited for studying cell architecture and organelle partitioning and interactions in intact cells (Müller *et al.*, 2012 ▸; Parkinson *et al.*, 2008 ▸). The contrast is directly dependent on the X-ray absorbance. Thus, carbon-rich organelles such as LDs absorb soft X-rays much more strongly than other organelles and become very distinctive (Seo *et al.*, 2017 ▸; Pérez-Berná *et al.*, 2016 ▸; Uchida *et al.*, 2011 ▸).

Second, we used synchrotron deep-ultraviolet (DUV) illumination allowing label-free imaging in living cells. DUV imaging was originally developed for label-free fluorescent analysis of biological samples. It has been successfully used to identify tissue organization in mammalian muscle fibres (Chagnot *et al.*, 2015 ▸) and in plant vascular tissue (Jamme *et al.*, 2013 ▸). Information was obtained at the micrometre scale and on fixed material but DUV imaging can also be used on living material at 110 nm spatial resolution (following the Rayleigh criterion) (Jamme *et al.*, 2010 ▸) with preservation of cell viability (Cinquin *et al.*, 2015 ▸). We investigated living-cell imaging using DUV transmittance (TRANS) imaging. This technique was developed on living mammalian cells for protein and DNA mapping based on their absorbance properties (Cheung *et al.*, 2011 ▸; Zeskind *et al.*, 2007 ▸). The technical challenge was to transpose these methodologies to living yeast. Unlike mammalian cells, yeasts are not adherent. Furthermore, yeasts are ten times smaller (5 µm versus 50 µm) than mammalian cells. Here, we provide LD ultrastructural data in a native environment using a new multimodal label-free imaging method.

## 2. Materials and methods   

### 2.1. Strains and culture conditions   

The strains used are listed in Table 1 ▸. Strains wild type (WT) W303, triple mutant (TM) Dga1^+^ and TM Are2^+^ are in kind gifted from Sten Stymne Laboratory (Sandager *et al.*, 2002 ▸). Cells were grown in rich medium supplemented with 2%(*w*/*v*) glucose. All cultures were grown in conical flasks containing 1/5 volume of medium and incubated at 28°C in an orbital shaker with an agitation rate of 200 r.p.m.

### 2.2. Cryo soft X-ray tomography   

4 µl of cell suspension (∼10^8^ cells ml^−1^) was deposited on plasma-cleaned quantifoil gold-finder grids and vitrified by plunge freezing using a Leica Automatic Plunge Freezer EM GP2. Cryo soft X-ray tomography data were collected at the MISTRAL beamline (ALBA Synchrotron, Barcelona, Spain) (Sorrentino *et al.*, 2015 ▸). The vitrified grids were transferred to the beamline under cryogenic and vacuum conditions. The datasets were acquired at an energy of 520 eV with an exposure time of 1 s, in a tilt range from −65 to 65° with 1° steps. Alignment of the tilted series was performed with *IMOD* (Kremer *et al.*, 1996 ▸). The final reconstructions were performed using *Tomo3D* (Agulleiro & Fernandez, 2015 ▸).

### 2.3. Full-field DUV microscopy   

Synchrotron DUV microscopy was performed at the DISCO beamline at the SOLEIL synchrotron radiation facility (Gif-sur-Yvette, France) on the TELEMOS microscope (Jamme *et al.*, 2013 ▸). Cells were deposited on quartz slides or a silicon surface and were observed under DUV excitation using a Zeiss AxioObserver Z1 through a 100× Zeiss ultrafluar glycerine immersion objective (numerical aperture of 1.25). The fluorescence was recorded under 275 nm excitation using a dichroic mirror at 300 nm (Omega Optical Inc.) and a 327–353 emission bandpass filter (Omega Optical Inc.) for tryptophan/tyrosine (TRP/TYR) auto-fluorescence, and under 340 nm excitation using a dichroic mirror at 410 nm (Omega Optical Inc.) and a 420–480 emission bandpass filter (Omega Optical Inc.) for DPH fluorescence with an exposure time of 5000 ms. The transmittance signal was recorded under 275 nm excitation after reflection in the silicon mirror using a 50/50 dichroic mirror with a 285/14 emission bandpass filter with an exposure time of 500 ms. *Z* vertical acquisitions were performed with a 300 nm step size following the Nyquist criterion. Images were recorded on a PIXIS 1024 BUV (Princeton) EM-CCD. For DPH staining, cells were incubated for 15 min at room temperature at the final concentrations recommended by the supplier (Thermo Fisher Scientific).

### 2.4. Point spread function deconvolution   

The Point spread function (PSF) describes how a point in the sample is imaged by microscope optics. The brightness of every point in the image is linearly related through convolution to the fluorescence of each point in the object. To calculate the deconvolution, the PSF of the whole system was recorded with calibrated fluorescent beads (0.17 µm TetraSpeck Molecular Probes, Life Technologies). This experimental PSF was then applied to the *Z* stack of images using a deconvolution classical maximum-likelihood estimation (Huygens software, SVI, The Netherlands). The *Z* step was 200 nm and the acquisition time was 10 s.

## 3. Results   

### 3.1. LD neutral lipid content can modify lipid core organization   

**Figure 1:**
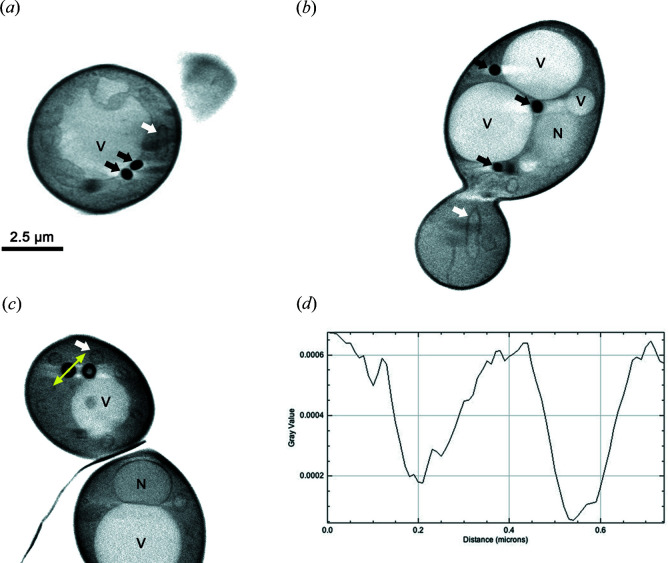
LD core heterogeneity revealed by cryo-SXT. WT, TM Dga1^+^ and TM Are2^+^ cells were vitrified on transmission electron microscopy gold grids using plunge freezing in liquid ethane, and observed under the transmission X-ray microscope of the MISTRAL beamline from the ALBA synchrotron. The tomographies were reconstructed from a series of tilt projections. The virtual reconstructed slices are from WT cell (*a*), TM Dga1^+^ cell (*b*) and TM Are2^+^ cell (*c*). (*d*) The LD intensity plot profile from the TM Are2^+^ cell was obtained along the line segment visualized by the yellow double arrow in panel (*c*). Nucleus (N), vacuole (V), LD (black arrow) and mitochondria (white arrow).

LD core ultrastructural organization was shown in *S. cerevisae* using SAXS on purified organelles but was not visible on cell thin sections using conventional electron microscopy (Czabany *et al.*, 2008 ▸). We investigated if LD lipid core arrangement could be observed using another imaging technique, cryo-SXT. In this case, vitrification is the only sample preparation step required and therefore the cells are in a close-to-native state. In *S. cerevisiae*, neutral lipid synthesis is dependent on four acyl-transferases, Dga1p and Lro1p for TAGs and Are1p and Are2p for SEs (Sandager *et al.*, 2002 ▸). We performed cryo-SXT on both the WT and TM strains with only one of these acyl­transferases, leading to cells producing only TAG (TM Dga1^+^) containing LDs and SE containing LDs (TM Are2^+^). 3D reconstructions were obtained from a series of tilted projections. The transmitted signal of the projections is directly related to the elemental concentration of the different features inside the cells. Virtual slices of the volumes revealed contrasted morphology of LDs in these three strains. LDs in WT [*n* = 20, Fig. 1 ▸(*a*), black arrows] and TM Dga1^+^ [*n* = 18, Fig. 1 ▸(*b*), black arrows] homogeneously absorbed X-rays with lipids inside the core. LDs in TM Are2^+^, containing only SEs, presented a core with a low-transmittance external black ring and a high-transmittance heart [*n* = 12, Fig. 1 ▸(*c*)], as confirmed by the LD intensity plot profile [Fig. 1 ▸(*d*)]. These results show that in SE-containing LDs the heart of the core does not contain as much carbon-rich material as the WT or TAG containing LDs, and SEs are confined at the periphery of the LD core.

### 3.2. LDs are high-absorbance organelles at 275 nm excitation   

**Figure 2:**
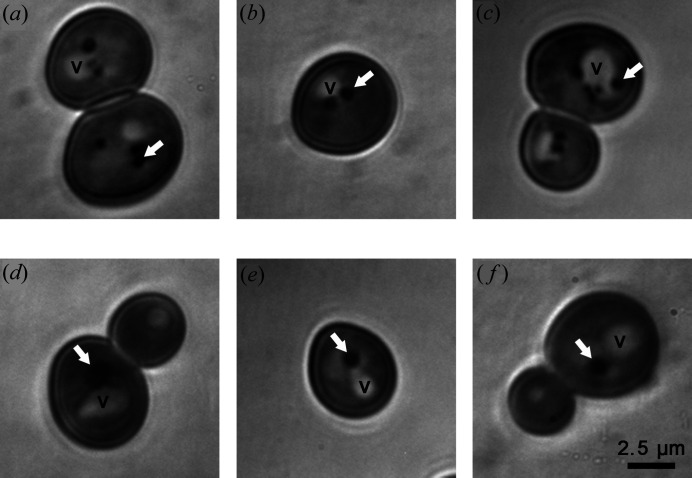
LD imaging using DUV transmittance microscopy. WT (*a*), (*b*), (*c*) and Sei1Δ (*d*), (*e*), (*f*) cells were observed using DUV transmittance microscopy. LD (white arrow), vacuole (V).

SEs in *S. cerevisiae* LDs are mainly ergosterol esters (Czabany *et al.*, 2008 ▸). To go further in SE partitioning in LD cores in living yeasts, we took advantage of an ergosterol absorbance spectra range between 240 and 300 nm with two main peaks at 270 and 280 nm, which is compatible with DUV illumination. We performed DUV transmittance (TRANS) imaging at 275 nm excitation on cells deposited on a reflective surface. The TRANS contrast generated by the absorbance properties of the cell material after reflection was collected. Observations were performed on cells containing LDs with distinguished morphologies to clearly identify LDs, as no previous literature was available on LD TRANS imaging. We chose WT cells with a few 250 nm average diameter LDs (Vindigni *et al.*, 2013 ▸) and Sei1Δ mutant cells with one supersized LD per cell (Fei *et al.*, 2008 ▸). We observed contrasting signals inside the cells. In WT cells [Figs. 2 ▸(*a*), 2(*b*) and 2(*c*)], we observed one white organelle with a high TRANS signal, multiple black round organelles with low TRANS signal and a medium TRANS signal corresponding to cytoplasm. In Sei1Δ mutant cells [Figs. 2 ▸(*d*), 2(*e*) and 2(*f*)], we observed white organelles with high TRANS signal and one black round organelle with a low TRANS signal. WT cell and Sei1Δ mutant-cell TRANS image comparison allowed the identification of vacuoles as high TRANS signal organelles and LDs as black round structures with low TRANS signal (Fig. 2 ▸, white arrows), probably because of LD high ergosterol content.

### 3.3. DUV TRANS imaging confirms lipid-core partitioning   

**Figure 3:**
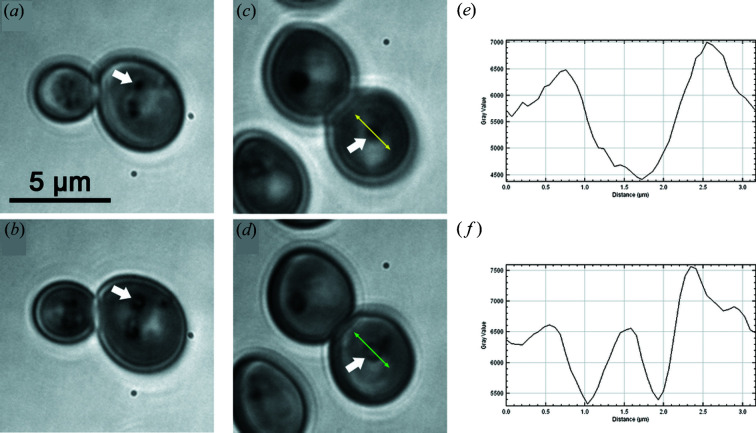
LD core heterogeneity revealed by DUV transmittance microscopy. WT (*a*), (*b*) and Sei1Δ (*c*), (*d*) cells were observed using DUV transmittance microscopy. 300 nm *Z* stacks were performed. LD surface (*a*), (*c*) and LD internal structure (*b*), (*d*) were imaged. LD intensity plot profiles from Sei1Δ LD surface (*e*) and Sei1Δ LD internal structure (*f*) were obtained along the line segments visualized by the yellow and green double arrows, respectively. LD (white arrow).

Image stacks obtained using cryo-SXT revealed the ultrastructural partitioning of SE-containing LDs. To investigate ultrastructural partitioning using TRANS imaging, we examined LDs at different *Z* positions in the cell. TRANS *Z* stacks revealed transmittance partitioning within LDs in WT cells [Figs. 3 ▸(*a*), and 3(*b*), white arrow] and Sei1Δ [Figs. 3 ▸(*c*) and 3(*d*), white arrow] mutant cells. LDs appear as low TRANS structures at the surface [Figs. 3 ▸(*a*) and 3(*c*)] and as donut-like structures with a low TRANS ring and a high TRANS heart from afar [Figs. 3 ▸(*b*) and 3(*d*)], as confirmed by the intensity plot profiles on LDs from Sei1Δ mutant cells at different *Z* stages [Figs. 3 ▸(*e*) and 3(*f*)]. We concluded that LD cores are made of a low TRANS peripheral layer compatible with an ergosterol-rich peripheral ring. These observations and the previous results obtained using cryo-SXT support a possible physiological partitioning of SEs at the periphery of LDs.

### 3.4. Combination of DUV TRP/TYR and TRANS imaging improves LD identification during label-free imaging   

**Figure 4:**
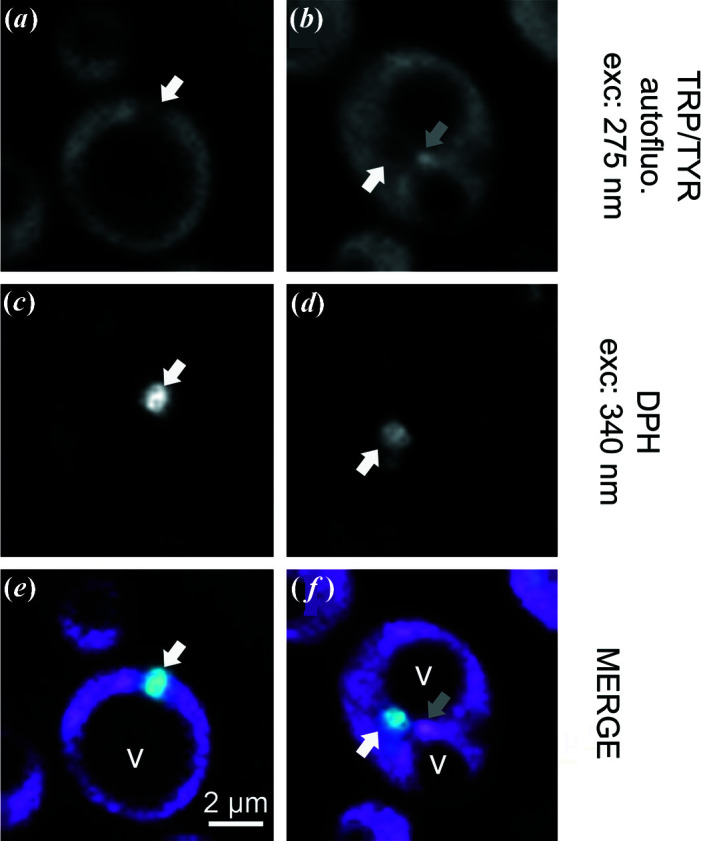
LD imaging using DUV fluorescence microscopy. Sei1Δ cells incubated with DPH stain were observed using DUV fluorescence microscopy at 275 nm and 340 nm excitations to obtain TRP/TYR autofluorescence (*a*, *b*) and LD localization (*c*, *d*), respectively. Merged images (*e*, *f*) were presented with TRP/TYR and DPH fluorescences as pink and blue signals, respectively. LD (white arrow), unidentified TRP/TYR fluorescent structure (grey arrow), vacuole (V).

Then, we investigated LD core lipid partitioning on WT cells and Sei1Δ mutant cells using DUV fluorescence imaging. Cells were deposited on a quartz slide and observed under DUV illumination at 275 nm with an emission bandpass filter ranging from 327 to 353 nm, corresponding mainly to TRP and TYR auto-fluorescence (Jamme *et al.*, 2013 ▸). The recorded image stacks were contaminated by out-of-focus contributions that can be reduced by image-restauration techniques (Van Kempen *et al.*, 1997 ▸). The visualization of contrasted signals inside the cells revealed protein-concentration heterogeneity at the subcellular level. Fluorescence microscopy showed a high TRP/TYR fluorescence for the cytoplasm and organelles with different levels of auto-fluorescence after illumination at 275 nm [Figs. 4 ▸(*a*) and 4(*b*)]. Some high TRP/TYR fluorescence bright structures, which remain to be identified, were observed [Fig. 4 ▸(*a*), grey arrow]. In contrast, some low TRP/TYR fluorescence organelles were visible. Identification of LDs was not trivial. The use of DPH, a vital neutral lipid probe with a maximum excitation compatible with DUV (340 nm), was necessary [Figs. 4 ▸(*c*) and 4(*d*)]. In Sei1Δ mutant cells, we identified LDs as low TRP/TYR organelles [Figs. 4 ▸(*e*) and 4(*f*), white arrows]. TRP/TYR fluorescence LDs were not distinguishable from the high TRP/TYR fluorescence signal of the cytoplasm in WT cells (data not shown) because of the smaller size of LDs. Vacuoles were identified as low TRP/TYR organelles because of their size and location inside the cell. The exploration of DUV fluorescence Sei1Δ mutant-cell *Z* stacks did not permit the observation of LD core partitioning, in contrast to DUV TRANS imaging.

**Figure 5:**
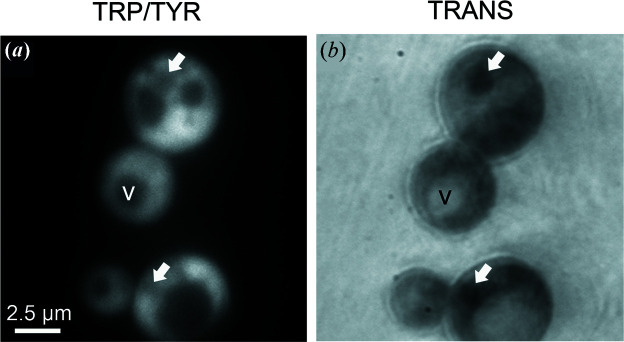
LD imaging using DUV multimodal microscopy. Sei1Δ cells were observed using TRP/TYR autofluorescence (*a*) and transmittance (*b*) imaging at 275 nm. LD (white arrow), vacuole (V).

We also explored the technical feasibility of performing DUV multimodal imaging on living *S. cerevisiae*. Sei1Δ mutant cells were deposited on a reflective silicon surface and subjected to 275 nm illumination. Fluorescence [Fig. 5 ▸(*a*)] and TRANS [Fig. 5 ▸(*b*)] images were sequentially recorded, and cell and organelle heterogeneity were observed.

TRANS and fluorescence DUV imaging gave different and complementary information on cell chemical content. Vacuoles and LDs were not distinguishable using TRP/TYR imaging. By combining multimodal images, we were able to clearly identify LDs as low TRP/TYR fluorescence and high TRANS organelles (Fig. 5 ▸, white arrows).

## 4. Discussion   

Our objective was to investigate the structure of LD lipid cores using nondestructive cell imaging such as cryo-SXT and DUV microscopy. DUV microscopy does not necessitate any special sample preparation whereas cell vitrification is necessary for cryo-SXT. This instantaneous freezing does not induce lipid-phase transition, such as suggested to be occurring during LD isolation procedures (Lei *et al.*, 2019 ▸). Thus, our sample preparation conditions are free of artefacts and therefore compatible with LD lipid core structural investigations.

Observation of LDs with contrasted neutral lipid content revealed a peculiar core structural organization in SE-rich LDs. Lowering TAG content in TM Are2^+^ LDs induces a SE structuration at the periphery of the LD core, without any SE colonization of the central part. Whereas, when TAGs are present, LDs appear homogeneously filled with carbon-dense lipids. LDs structural features seem not to be restricted to yeast models and have previously been visualized, for instance, in a subclass of LDs in fibroblast cells using cryo-SXT (Groen *et al.*, 2019 ▸). The nature of the central low carbon-density ‘hole’ in TM Are2^+^ LDs remains a mystery. Proteins were also found inside the LD lipid core (Robenek *et al.*, 2011 ▸; Cermelli *et al.*, 2006 ▸). The presence of proteins in the low carbon-density heart of the SE-rich LD core could be investigated, for instance, by looking at the nitro­gen absorption edge using soft X-ray spectroscopic imaging at the MISTRAL beamline.

Structural changes induced by modification of the TAG/SE ratio are documented for lipoproteins. Higher SE content in low-density lipoproteins (LDLs) increases the lipid-phase transition temperature, favours structural changes at biological temperatures (from melted core to crystalized/organized core with probable lipid-phase separation), and modifies shape and protein association properties of the LDL surface (Lei *et al.*, 2019 ▸; Sherman *et al.*, 2003 ▸). We could hypothesize that the same behaviour could occur for cytoplasmic LDs as we observed notable structural differences between TAG-containing LD cores and LD cores with SE only. In our case, this structural LD core modification was not associated with intracellular modification of LD shape, but we did not exclude possible modification of LD surface properties. Modification of LD structure induced by neutral lipid content could be a way to modify the physiological state and dynamics of cytoplasmic LDs, as observed for LDL. Recently, LD structural changes in HeLa cells during the cell cycle were observed using cryo-FIB. These data support such models of LD dynamic control (Mahamid *et al.*, 2019 ▸).

DUV light provided by the DISCO beamline enabled imaging of organelles in the living unicellular yeast. As the resolution is directly related to the wavelength, working in DUV makes it possible to naturally increase the lateral resolution to 110 nm, allowing visualization of structures like contact zones between two organelles or intra-organelle structural partitioning. DUV LD TRANS revealed LD core chemical heterogeneity with a low-transmittance ring and a high-transmittance central heart at 275 nm excitation. This shows that LDs are well organized structures in living cells.

Yeast LDs contain TAGs and ergosterol esters. The ergo­sterol absorbance spectra range was between 240 and 300 nm with two main peaks at 270 and 280 nm. With 275 nm excitation, we recorded transmittance signals for ergosterol and by-products present in cellular membranes, but also in LDs. In WT yeasts, like in Sei1Δ strains (Fei *et al.*, 2011 ▸), the LDs mainly contain ergosterol esters and TAGs in the same proportion. Unlike ergosterol, TAGs do not absorb at 275 nm. Consequently, the low-transmittance ring and the high-transmittance heart could correspond to ergosterol and TAG-containing structures, respectively. Such LD structural organization was observed for purified LDs using SAXS (Czabany *et al.*, 2008 ▸) and for LDs in vitreous lamellae using cryo-FIB (Mahamid *et al.*, 2019 ▸). Here, we strengthen the concentric layer model of LD core structural organization using the fast-imaging technique in living cells.

We also constructed dedicated procedures for the combination of TRANS and TRP/TYR auto-fluorescence imaging on living cells. This DUV multimodal imaging gave different and complementary information on cell chemical content. This technique improves identification of intracellular compartments at the subcellular level based on their dual chemical-imaging signature. This powerful and original multimodal-imaging technique paves the way for the constitution of an organelle atlas in living cells.

In conclusion, both cryo-SXT and DUV imaging provide structural and chemical information at different scales. By combining these synchrotron techniques, we integrated data from the submicrometre scale to the micrometre scale. In particular, for LD structure, the organelle ultrastructure has been investigated in connection with the cellular organization thanks to the scales covered by the two techniques. Each of these synchrotron techniques also present unique assets. Cryo-SXT provides a resolution compatible with the fine ultrastructure of cellular compartments (Groen *et al.*, 2019 ▸), while DUV imaging offers dynamics of biological processes such as cellular divisions, a follow up of the intracellular fate of external molecules (Vergalli *et al.*, 2018 ▸), and abiotic and biotic stress responses. 

## Figures and Tables

**Table 1:** Yeast strains used in this study

Name	Strain	Relevant genotype	Experiment	Source and reference
WT	W303	MATa	Cryo-SXT	Sten Stymne Laboratory (Sandager *et al.*, 2002 ▸)
TM Dga1^+^	W303	MATα Are1Δ::HIS3, Are2Δ::LEU2, Lro1Δ::URA3	Cryo-SXT	Sten Stymne Laboratory (Sandager *et al.*, 2002 ▸)
TM Are2^+^	W303	MATα Are1Δ::HIS3 Dga1Δ::KanMX4 Lro1Δ::URA3	Cryo-SXT	Sten Stymne Laboratory (Sandager *et al.*, 2002 ▸)
WT	BY4741	MATa	DUV	Euroscarf
Sei1Δ	BY4741	MATa Ylr404wΔ::KanMX4	DUV	Euroscarf

## References

[bb1] Agulleiro, J. I. & Fernandez, J. J. (2015). *J. Struct. Biol.* **189**, 147–152. 10.1016/j.jsb.2014.11.009 25528570

[bb2] Bouchez, I., Pouteaux, M., Canonge, M., Genet, M., Chardot, T., Guillot, A. & Froissard, M. (2015). *Biol. Open*, **4**, 764–775. 10.1242/bio.20148615 PMC4571102 25948753

[bb3] Cermelli, S., Guo, Y., Gross, S. P. & Welte, M. A. (2006). *Curr. Biol.* **16**, 1783–1795. 10.1016/j.cub.2006.07.062 16979555

[bb4] Chagnot, C., Vénien, A., Peyrin, F., Jamme, F., RéFrégiers, M., Desvaux, M. & Astruc, T. (2015). *Analyst*, **140**, 4189–4196. 10.1039/c5an00172b 25912941

[bb5] Cheng, J. L., Fujita, A., Ohsaki, Y., Suzuki, M., Shinohara, Y. & Fujimoto, T. (2009). *Histochem. Cell Biol.* **132**, 281–291. 10.1007/s00418-009-0615-z 19557427

[bb6] Cheung, M. C., Evans, J. G., McKenna, B. & Ehrlich, D. J. (2011). *Cytometry A*, **79**, 920–932. 10.1002/cyto.a.21111 PMC3199293 21796773

[bb7] Cinquin, B., Maigre, L., Pinet, E., Chevalier, J., Stavenger, R. A., Mills, S., RéFrégiers, M. & Pagès, J. M. (2015). *Sci. Rep.* **5**, 17968. 10.1038/srep17968 PMC4675965 26656111

[bb8] Czabany, T., Wagner, A., Zweytick, D., Lohner, K., Leitner, E., Ingolic, E. & Daum, G. (2008). *J. Biol. Chem.* **283**, 17065–17074. 10.1074/jbc.M800401200 18430725

[bb9] Dyer, J. M., Stymne, S., Green, A. G. & Carlsson, A. S. (2008). *Plant J.* **54**, 640–655. 10.1111/j.1365-313X.2008.03430.x 18476869

[bb10] Fei, W., Shui, G., Gaeta, B., Du, X., Kuerschner, L., Li, P., Brown, A. J., Wenk, M. R., Parton, R. G. & Yang, H. (2008). *J. Cell Biol.* **180**, 473–482. 10.1083/jcb.200711136 PMC2234226 18250201

[bb11] Fei, W., Shui, G., Zhang, Y., Krahmer, N., Ferguson, C., Kapterian, T. S., Lin, R. C., Dawes, I. W., Brown, A. J., Li, P., Huang, X., Parton, R. G., Wenk, M. R., Walther, T. C. & Yang, H. (2011). *PLoS Genet.* **7**, e1002201. 10.1371/journal.pgen.1002201 PMC3145623 21829381

[bb12] Gao, Q. & Goodman, J. M. (2015). *Front. Cell. Dev. Biol.* **3**, 49. 10.3389/fcell.2015.00049 PMC4533013 26322308

[bb13] Groen, J., Conesa, J. J., Valcárcel, R. & Pereiro, E. (2019). *Biophys. Rev.* pp. 611–619. 10.1007/s12551-019-00567-6 PMC6682196 31273607

[bb15] Hashemi, H. F. & Goodman, J. M. (2015). *Curr. Opin. Cell Biol.* **33**, 119–124. 10.1016/j.ceb.2015.02.002 PMC4380764 25703629

[bb16] Jacquier, N., Choudhary, V., Mari, M., Toulmay, A., Reggiori, F. & Schneiter, R. (2011). *J. Cell Sci.* **124**, 2424–2437. 10.1242/jcs.076836 21693588

[bb17] Jamme, F., Kascakova, S., Villette, S., Allouche, F., Pallu, S., Rouam, V. & RéFrégiers, M. (2013). *Biol. Cell*, **105**, 277–288. 10.1111/boc.201200075 23517500

[bb18] Jamme, F., Villette, S., Giuliani, A., Rouam, V., Wien, F., Lagarde, B. & RéFrégiers, M. (2010). *Microsc. Microanal.* **16**, 507–514. 10.1017/S1431927610093852 20738889

[bb19] Kory, N., Farese, R. V. Jr & Walther, T. C. (2016). *Trends Cell Biol.* **26**, 535–546. 10.1016/j.tcb.2016.02.007 PMC4976449 26995697

[bb20] Kremer, J. R., Mastronarde, D. N. & McIntosh, J. R. (1996). *J. Struct. Biol.* **116**, 71–76. 10.1006/jsbi.1996.0013 8742726

[bb21] Lei, D., Yu, Y., Kuang, Y. L., Liu, J., Krauss, R. M. & Ren, G. (2019). *Biochim. Biophys. Acta Mol. Cell. Biol. Lipids*, **1864**, 260–270. 10.1016/j.bbalip.2018.12.004 PMC6409128 30557627

[bb22] Mahamid, J., Tegunov, D., Maiser, A., Arnold, J., Leonhardt, H., Plitzko, J. M. & Baumeister, W. (2019). *Proc. Natl Acad. Sci. USA*, **116**, 16866–16871. 10.1073/pnas.1903642116 PMC6708344 31375636

[bb23] Müller, W. G., Bernard Heymann, J., Nagashima, K., Guttmann, P., Werner, S., Rehbein, S., Schneider, G. & McNally, J. G. (2012). *J. Struct. Biol.* **177**, 179–192. 10.1016/j.jsb.2011.11.025 PMC3288423 22155291

[bb24] Ohsaki, Y., Suzuki, M. & Fujimoto, T. (2014). *Chem. Biol.* **21**, 86–96. 10.1016/j.chembiol.2013.08.009 24239006

[bb25] Olzmann, J. A. & Carvalho, P. (2019). *Nat. Rev. Mol. Cell Biol.* **20**, 137–155. 10.1038/s41580-018-0085-z PMC6746329 30523332

[bb26] Parkinson, D. Y., McDermott, G., Etkin, L. D., Le Gros, M. A. & Larabell, C. A. (2008). *J. Struct. Biol.* **162**, 380–386. 10.1016/j.jsb.2008.02.003 PMC2505111 18387313

[bb27] Pérez-Berná, A. J., Rodríguez, M. J., Chichón, F. J., Friesland, M. F., Sorrentino, A., Carrascosa, J. L., Pereiro, E. & Gastaminza, P. (2016). *ACS Nano*, **10**, 6597–6611. 10.1021/acsnano.6b01374 27328170

[bb28] Pyc, M., Cai, Y., Greer, M. S., Yurchenko, O., Chapman, K. D., Dyer, J. M. & Mullen, R. T. (2017). *Trends Plant Sci.* **22**, 596–609. 10.1016/j.tplants.2017.03.012 28454678

[bb29] Robenek, H., Buers, I., Robenek, M. J., Hofnagel, O., Ruebel, A., Troyer, D. & Severs, N. J. (2011). *J. Lipids*, **2011**, 409371. 10.1155/2011/409371 PMC3068475 21490801

[bb30] Sandager, L., Gustavsson, M. H., Ståhl, U., Dahlqvist, A., Wiberg, E., Banas, A., Lenman, M., Ronne, H. & Stymne, S. (2002). *J. Biol. Chem.* **277**, 6478–6482. 10.1074/jbc.M109109200 11741946

[bb31] Seo, A. Y., Lau, P. W., Feliciano, D., Sengupta, P., Gros, M. A. L., Cinquin, B., Larabell, C. A. & Lippincott-Schwartz, J. (2017). *eLife*, **6**, 27. 10.7554/eLife.21690 PMC5407857 28394250

[bb32] Sherman, M. B., Orlova, E. V., Decker, G. L., Chiu, W. & Pownall, H. J. (2003). *Biochemistry*, **42**, 14988–14993. 10.1021/bi0354738 14674775

[bb33] Sorrentino, A., Nicolás, J., Valcárcel, R., Chichón, F. J., Rosanes, M., Avila, J., Tkachuk, A., Irwin, J., Ferrer, S. & Pereiro, E. (2015). *J. Synchrotron Rad.* **22**, 1112–1117. 10.1107/S1600577515008632 26134819

[bb34] Tauchi-Sato, K., Ozeki, S., Houjou, T., Taguchi, R. & Fujimoto, T. (2002). *J. Biol. Chem.* **277**, 44507–44512. 10.1074/jbc.M207712200 12221100

[bb35] Thiam, A. R. & Beller, M. (2017). *J. Cell Sci.* **130**, 315–324. 10.1242/jcs.192021 28049719

[bb36] Uchida, M., Sun, Y., McDermott, G., Knoechel, C., Le Gros, M. A., Parkinson, D., Drubin, D. G. & Larabell, C. A. (2011). *Yeast*, **28**, 227–236. 10.1002/yea.1834 PMC3404734 21360734

[bb37] Van Kempen, G. M. P., Van Vliet, L. J., Verveer, P. J. & Van Der Voort, H. T. M. (1997). *J. Microsc.* **185**, 354–365.

[bb38] Vergalli, J., Dumont, E., Pajović, J., Cinquin, B., Maigre, L., Masi, M., RéFrégiers, M. & Pagés, J. M. (2018). *Nat. Protoc.* **13**, 1348–1361. 10.1038/nprot.2018.036 29773906

[bb39] Vindigni, J. D., Wien, F., Giuliani, A., Erpapazoglou, Z., Tache, R., Jagic, F., Chardot, T., Gohon, Y. & Froissard, M. (2013). *Biochim. Biophys. Acta*, **1828**, 1881–1888. 10.1016/j.bbamem.2013.04.009 23603223

[bb40] Welte, M. A. (2015). *Curr. Biol.* **25**, R470–R481. 10.1016/j.cub.2015.04.004 PMC4452895 26035793

[bb41] Zeskind, B. J., Jordan, C. D., Timp, W., Trapani, L., Waller, G., Horodincu, V., Ehrlich, D. J. & Matsudaira, P. (2007). *Nat. Methods*, **4**, 567–569. 10.1038/nmeth1053 17546037

